# An Improvement of Pose Measurement Method Using Global Control Points Calibration

**DOI:** 10.1371/journal.pone.0133905

**Published:** 2015-07-24

**Authors:** Changku Sun, Pengfei Sun, Peng Wang

**Affiliations:** 1 State Key Laboratory of Precision Measuring Technology and Instruments, Tianjin University, Tianjin, China; 2 Science and Technology on Electro-optic Control Laboratory, Luoyang Institute of Electro-optic Equipment, Luoyang, China; Nankai University, CHINA

## Abstract

During the last decade pose measurement technologies have gained an increasing interest in the computer vision. The vision-based pose measurement method has been widely applied in complex environments. However, the pose measurement error is a problem in the measurement applications. It grows rapidly with increasing measurement range. In order to meet the demand of high accuracy in large measurement range, a measurement error reduction solution to the vision-based pose measurement method, called Global Control Point Calibration (GCPC), is proposed. GCPC is an optimized process of existing visual pose measurement methods. The core of GCPC is to divide the measurement error into two types: the control point error and the control space error. Then by creating the global control points as well as performing error calibration of object pose, the two errors are processed. The control point error can be eliminated and the control space error is minimized. GCPC is experimented on the moving target in the camera’s field of view. The results show that the RMS error is 0.175° in yaw angle, 0.189° in pitch angle, and 0.159° in roll angle, which demonstrate that GCPC works effectively and stably.

## Introduction

Detecting the rigid transformation of images into known geometry, namely the pose measurement, is one of the central problems in aircraft inflight refueling, spacecraft docking, and comprehensive helmet mounted display [1–3]. In the aircraft control during aerial refueling, it is commonly used to provide accurate relative position measurements to the controller of unmanned air vehicle [4]. In spacecraft docking, pose measurement is central to the positioning of the docking assembly, and accomplished with the assistance of artificial markers or natural markers on the spacecraft [5]. In comprehensive helmet mounted display, it plays a significant role in combining the pose of helmet with direction of the weapon or sensor [6].

There are many technologies such as magnetic, ultrasonic, and mechanical ones in pose measurement fields [7, 8]. Vision-based pose measurement technology stands out for its excellent anti-jamming capability and adaptability in harsh environments.

According to the difference of track target, vision-based tracking technology is divided into the following two categories [3]. The two categories are distinguished by markers, such as planar marker or point marker. One type is planar marker. It uses the perpendicular line segments, parallel line segments, intersection of the adjacent lines, and asymmetry of the cutting-off corner as track target [9, 10]. Planar marker is rarely used in practical application, because it requires both high manufacturing precision and rigid geometric constraints. Hence, the natural marker of objects replaces the manual planar marker. The other type is point marker. Each marker of this type presents one point correspondence between the scene and the image. Point marker such as circular marker is introduced, because the appearance of circular patterns is relatively invariant under perspective distortion and because their centroid provides a stable 2D position that can easily be determined with sub-pixel accuracy. The widely used pose measurement method based on point marker is known in the literature as the Perspective-n-Point(PnP) problem, whose objective is to measure the object pose based on image of known point markers [11]. There are lots of papers researching on the PnP problem, and the solutions to the problem are classified into two types: polynomial method and iterative method [12–14]. The former formulates a fourth to eighth order polynomial system by using three to five correspondences of the observed points to solve the PnP problem. And the iterative method regards the PnP problem as an optimization problem of the affine invariant cost function. The solutions are tested by practical applications, confirming both of the two methods need precision enhancement. A deep analysis has been performed on the pose measurement method, and a regular error is found during the measurement process. Michael D. Grossberg and Shree K. Nayar find an object space error through the analysis of linear perspective projection in [15]. The object space error is defined as the distance between the world point and the projection of this point onto the line of sight. In [16], the object space error is introduced into the PnP problem. Gerald Schweighofer and Axel Pinz recast the PnP problem as a minimization function by the given world points and their measurement in a camera, and the objective function is the minimum costs of the points. Furthermore, Hatem Hmam and Jijoong Kim formulate the object space error as a semidefinite positive relaxation(SDR) program. A convex relaxation is employed to solve the SDR in [17]. There are still other papers studying on the image error of point marker. The image error stems from the difference between the real centroid and the ideal centroid. Three methods of feature point tracking are proposed in [18]. They are compared in terms of accuracy and stability, but ignoring the impact on pose measurement. Bart Ons et al. find the adverse impact and propose the visual anisotropy of computational model [19]. The illusory orientation bias of three Gaussian Luminance ellipses are discussed, and furthermore, it is proved that the extracted center of bright ellipse and the physical angular coordinates are not coincident.

According to the methods above, the object space error is produced from the process for the theoretical imaging model approximate to the perspective projection model, and the image error is influenced by the brightness distribution and the edge of feature point. The current papers focus on the reduction of measurement error through more accurate parameters of imaging model or better extraction of centroid [15–19]. However, those two methods are not powerful enough to eliminate the inconformity. As the sources and the influence factors of the two errors are varied, they are redistributed without considering the nature in this paper. The two errors are redefined as two types: the control point error and the control space error. Then by creating the global control points as well as performing error calibration of object pose, the two errors are processed. The control point error is the measurement error between the initial reference point and the global control point while the control space error is the measurement error between the measuring point and the corresponding global control point. The first error is the primary source of the measurement error and eliminated by using the standard reference data of moving space. The other error is reduced by decreasing the control range of global control point. According to the analysis above, the Global Control Point Calibration(GCPC) is proposed to create the global control points and calibrate the measurement error of object pose.

## Description of System

**Fig 1:**
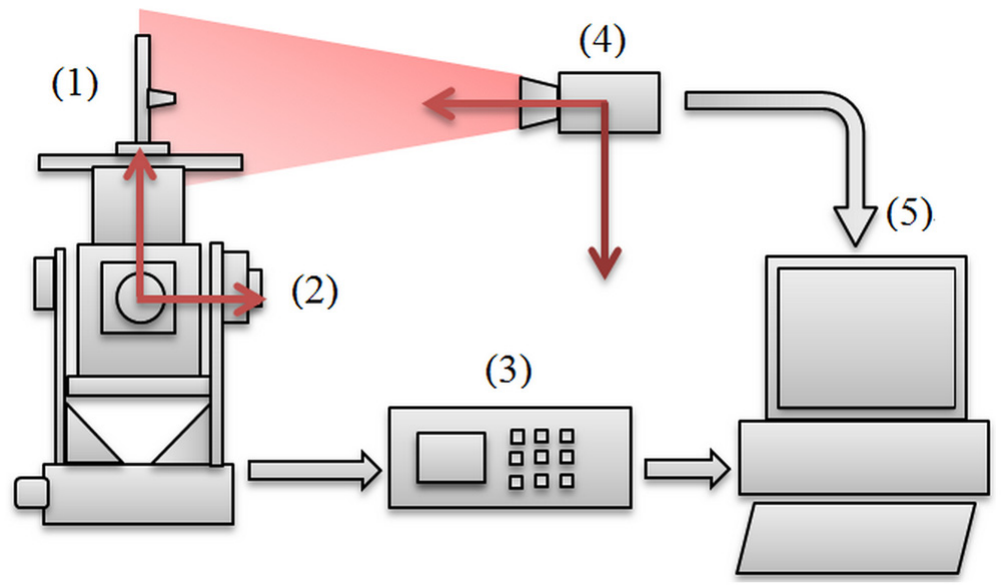
System Diagram.

The proposed schematic diagram of GCPC for pose measurement is shown in Fig 1. The system consists of (1) a target, (2) a three-axis turntable, (3) a turntable control box, (4) a camera, and (5) a computer.

The devices work in the following way: The target is fixed on the three-axis turntable which is controlled by the turntable control box; the image of rotating target is captured by the camera while the three standard Euler angles of turntable are read by the control box; both of the two sets of data are transmitted into the computer simultaneously.

The initial coordinate of feature point is calculated as the image of rotating target is transmitted into the computer. The algorithm of point coordinate is the Pose from Orthography and Scaling with Iterations(POSIT)[20]. POSIT is a classical algorithm and approved by scholars, companies, and defense [21, 22].

**Fig 2:**
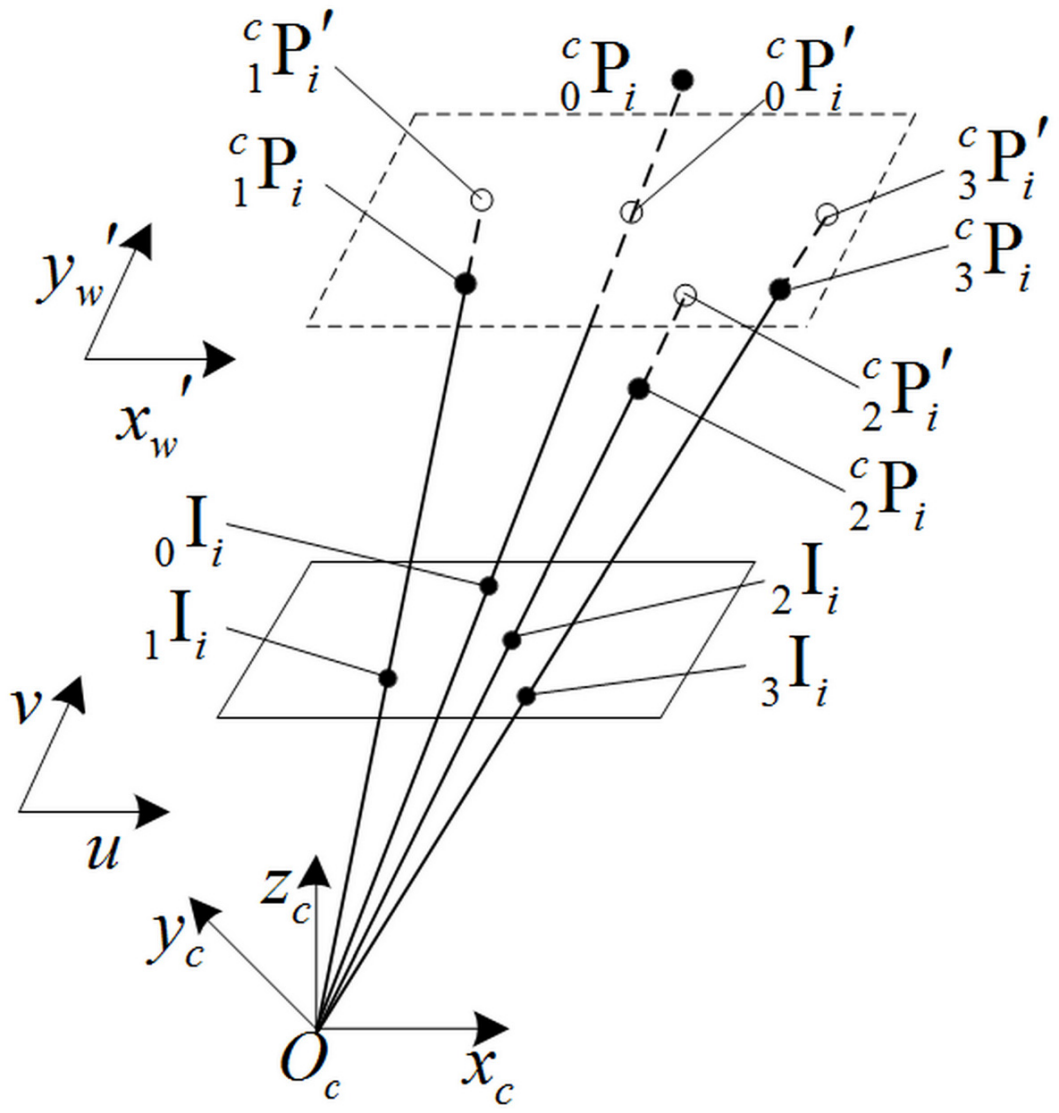
Geometric interpretation of POSIT. The pinhole camera with the center of projection at *O*_*c*_, optical axis aligned with *O*_*c*_, image plane *uv* at a distance *f* from *O*_*c*_. The origin of object coordinate system at Pmci, *m* = 0,1,2,3, *i* is the number of object’s position, *c* is the camera coordinate system, _*m*_I_*i*_ is the corresponding image point set, Pmci′ is the corresponding orthographic projecting point set.

The principle of POSIT is shown as Fig 2. The feature points Pmci have the depth zmci while the orthographic projecting points Pmci′ have the same depth zmci′. The cost function is formed as:

{εx=f∑m=03|xmci/zmci−xmci/zmci′|εy=f∑m=03|ymci/zmci−ymci/zmci′| (1)

where *f* is the focal length of camera, zmci′ is the average depth of feature points Pmci(*m* = 0, 1, 2, 3). In scaled orthographic projection, the image of a point Pmci is x′=xmci/zmci′ and y′=ymci/zmci′ while in perspective projection the image of that is x=xmci/zmci and y=ymci/zmci. The coordinates (xmci,ymci,zmci) of feature point Pmci is calculated in Eq (2) as *ε*_*x*_ and *ε*_*y*_ below a threshold value or when the run times reach the limit.

Pmci=[RcojTcoj01]Pmoji (2)

where *oj* is the object coordinate system, [RcojTcoj01] is the matrix which corresponds to the minimum of *ε*_*x*_ and *ε*_*y*_.

## The Measurement Reference of GCPC

**Fig 3:**
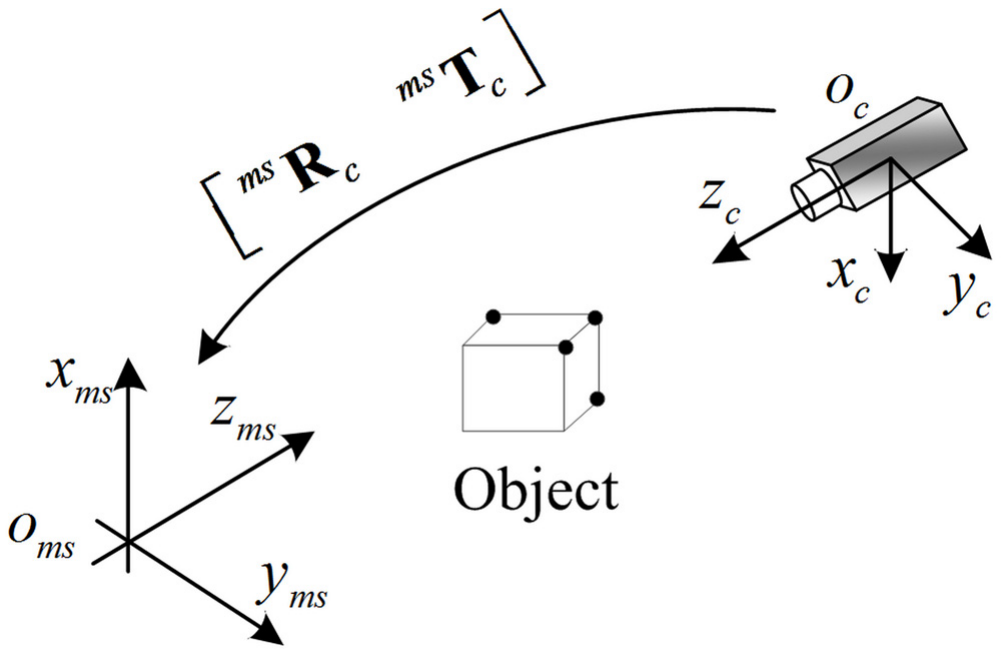
Coordinate systems of object moving. O_ms_-X_ms_Y_ms_Z_ms_ represents the measurement reference.

Through Eqs (1) and (2), the spatial coordinate of Pmci, based on the camera coordinate system, is obtained. The following step is to transform from Pmci to Pmmsi. Pmmsi is the spatial coordinate which is based on the measurement reference. Furthermore, both the creation of global control points and the performance of error calibration are conducted in measurement reference. The relationship of the measurement reference to the other coordinate system are shown in Fig 3.

The relationship between the O_c_-X_c_Y_c_Z_c_ and the O_ms_-X_ms_Y_ms_Z_ms_ should be described as:

Pmmsi=[RmscTmsc01]Pmci (3)

The matrix Rmsc and the vector Tmsc are described as:

{Rmsc=[h1h2h3]TTmsc=[uvw]T (4)

where **h**_1_ and **h**_2_ are respectively the unit direction vector of o_ms_-x_ms_ and o_ms_-y_ms_, **h**_3_ = **h**_1_ × **h**_2_. The (*u*, *v*, *w*) is the vector $\vec{o_{c}o_{ms}}$.

**Fig 4:**
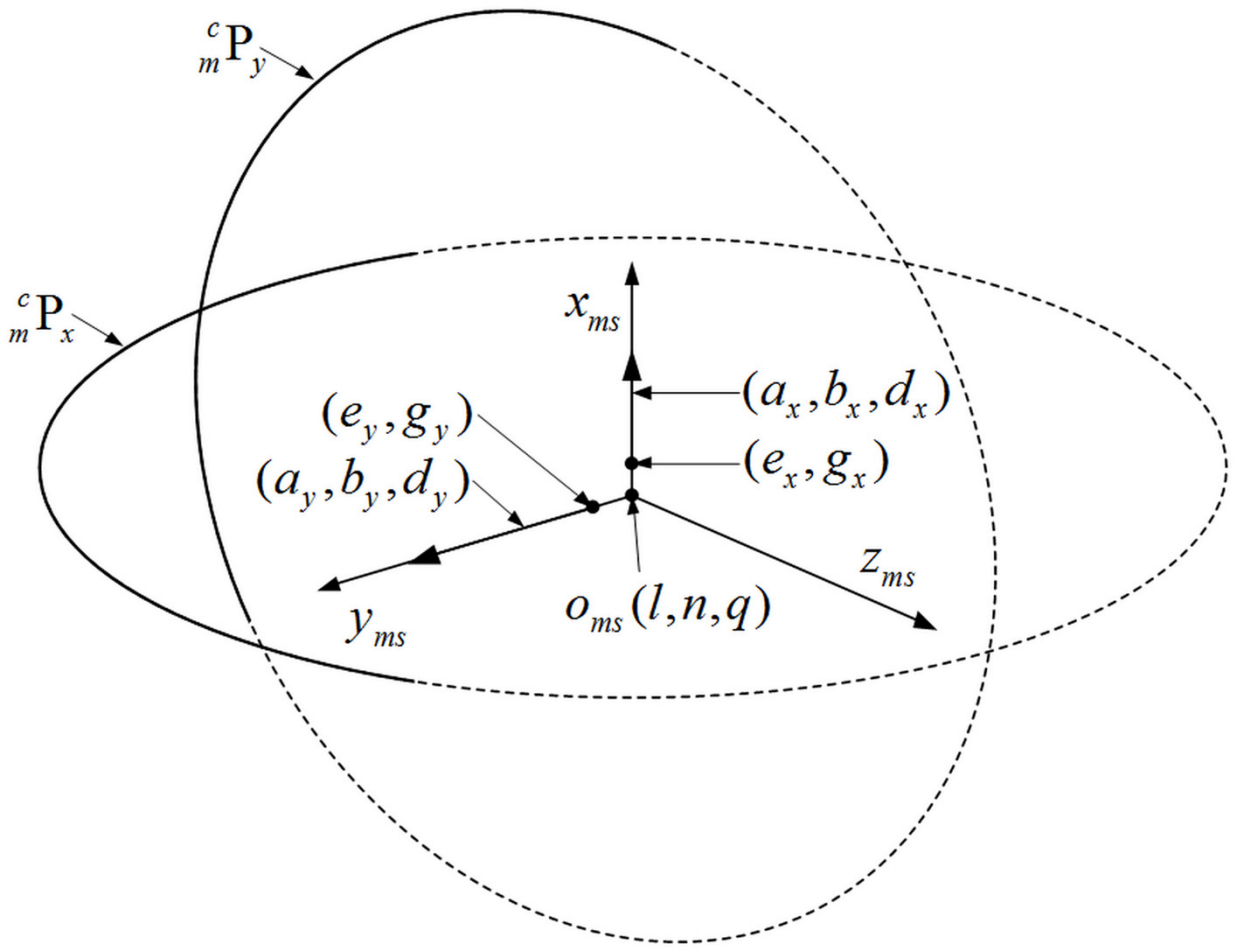
The measurement reference of GCPC. The (*a*_*x*_, *b*_*x*_, *d*_*x*_) and (*e*_*x*_, *g*_*x*_) corresponds to the Pmcx while the (*a*_*y*_, *b*_*y*_, *d*_*y*_) and (*e*_*y*_, *g*_*y*_) corresponds to the Pmcy. The two dotted circles are determined by the two solid arcs respectively.

The point sets Pmcx and Pmcy are selected respectively to establish o_ms_-x_ms_ and o_ms_-y_ms_. Both of them only rotate around an axis. *x* is the number of object’s position which rotates around the o_ms_-x_ms_ and *y* is the number of object’s position which rotates around the o_ms_-y_ms_. Taking the point sets into Eq (5) [23, 24]:

{ε2=∑i(axmci+bymci+czmci+d)2(a)ζ2=∑i[(xmci−e)2+(ymci−g)2−r2]2(b) (5)

where Eq 5(a) is the equation of plane fitting, the coefficient (*a*, *b*, *d*) is the direction vector of plane; 5(b) is the equation of circle fitting, point (*e*, *g*) is the anchor point of axis which locates in the fitting plane, *r* is the circle radius. The unit vectors of o_ms_-x_ms_ and o_ms_-y_ms_ are determined through Eq (5). The fitting process is described in Fig 4. The two dotted circles are determined by Eq 5(a), and they locate in the planes determined by Eq 5(b) respectively.

As the point sets Pmcx, Pmcy, and Pmcz rotate around the axis o_ms_-x_ms_, o_ms_-y_ms_, and o_ms_-z_ms_ respectively, they share a center of rotation theoretically. As the trajectories of them are non-coplanar arcs, a sphere fitting is adopted to describe the arcs. Taking the three point sets into the following sphere fitting equation, the sphere center is the shared center of roation [25].

Y=UV (6)

where Y=[xmc12+ymc12+zmc12xmc22+ymc22+zmc22⋮xmci2+ymci2+zmci2],U=[2xmc12ymc12zmc112xmc22ymc22zmc21⋮⋮⋮⋮2xmci2ymci2zmci1],$V=\left[\begin{array}{c} l\\ n\\ q\\ h^{2}-l^{2}-n^{2}-q^{2} \end{array}\right]$, (*l*, *n*, *q*) is the sphere center, *h* is the sphere radius.

As the measurement reference O_ms_-X_ms_Y_ms_Z_ms_ is established, the coordinate (xmmsi,ymmsi,zmmsi) of feature point Pmmsi is obtained.

## The Principle of GCPC

**Fig 5:**
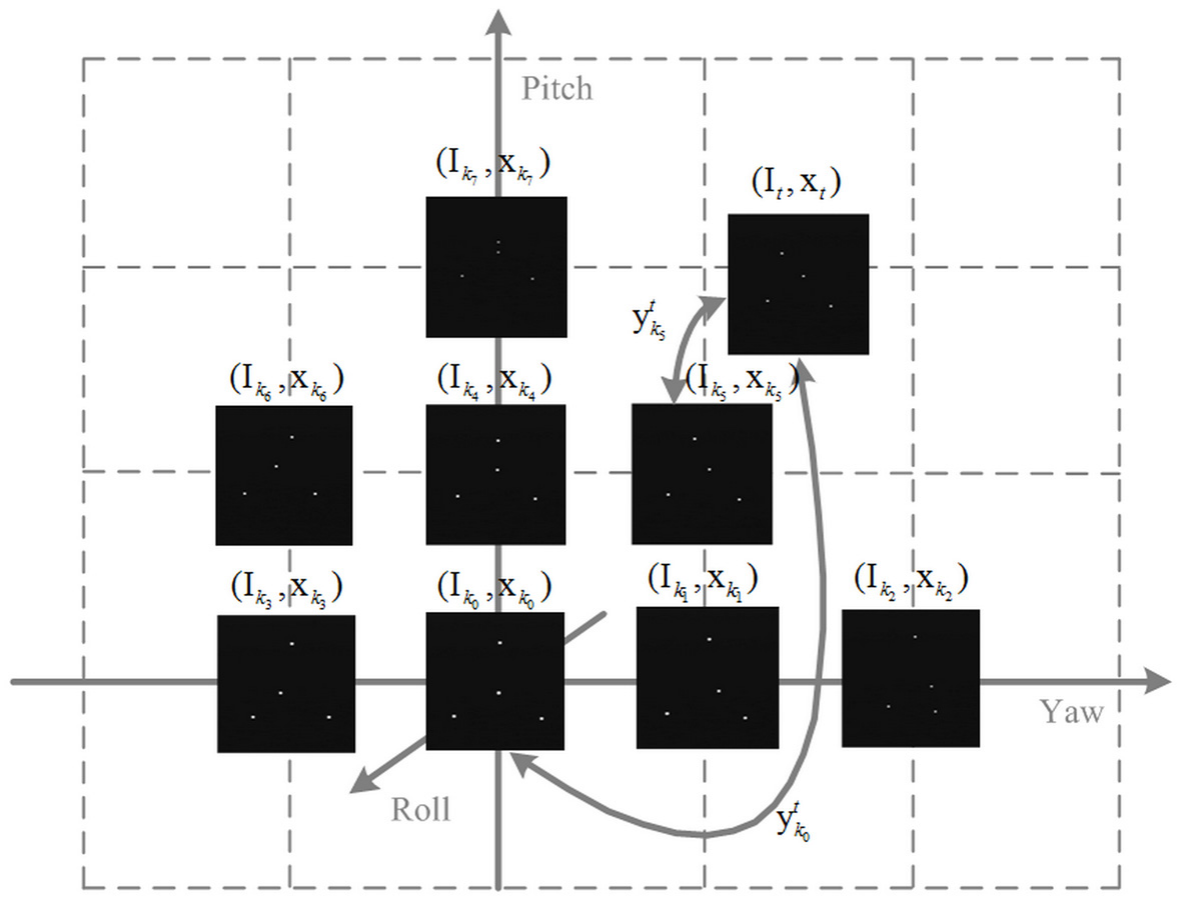
The schematic diagram of GCPC. FM_i_ keeps control of the space around it. *k*_*i*_ and *t* respectively represent the number of control point and measuring point. y*^j^_i_* is the measured value of pose vector between FM_*i*_ and FM_*j*_.

The information of object pose in the measurement reference is formally defined as:

$${\text{FM}}_{i}=\{{\text{I}}_{i},{\text{x}}_{i}\}$$ (7)

where the object pose is represented by I_*i*_ and x_*i*_ which are respectively the image feature and the standard pose vector. According to the expression, GCPC is organized as the following overview Fig 5.

The control point error is the measurement error between the global control point ${\text{FM}}_{k_{i}}$ and the reference point ${\text{FM}}_{k_{0}}$ while the control space error is the measurement error between the measuring point FM_*t*_ and the corresponding control point ${\text{FM}}_{k_{i}}$. The measured pose vector ${\text{x}}_{t}{}^{\prime }$ is obtained in two ways:

$${\text{x}}_{t}{}^{\prime }={\text{y}}_{k_{0}}^{t}$$ (8)

$${\text{x}}_{t}{}^{\prime }=({\text{x}}_{k_{5}}^{}-{\text{x}}_{k_{0}}^{})+{\text{y}}_{k_{5}}^{t}$$ (9)

In Eq (8), ${\text{y}}_{k_{0}}^{t}$ is the directly measured value of x_*t*_, and shows both the control point error and the control space error. In Eq (9), ${\text{y}}_{k_{5}}^{t}$ is the measured value of $\left({\text{x}}_{t}^{}-{\text{x}}_{k_{5}}^{}\right)$, which contains the control space error of ${\text{FM}}_{k_{5}}^{}$. $\left({\text{x}}_{k_{5}}^{}-{\text{x}}_{k_{0}}^{}\right)$ is the standard pose vector between the reference point ${\text{FM}}_{k_{0}}^{}$ and the control point ${\text{FM}}_{k_{5}}^{}$. GCPC optimizes the object pose FM_*t*_ by using Eq (9).

## The Implementation of GCPC

**Fig 6:**
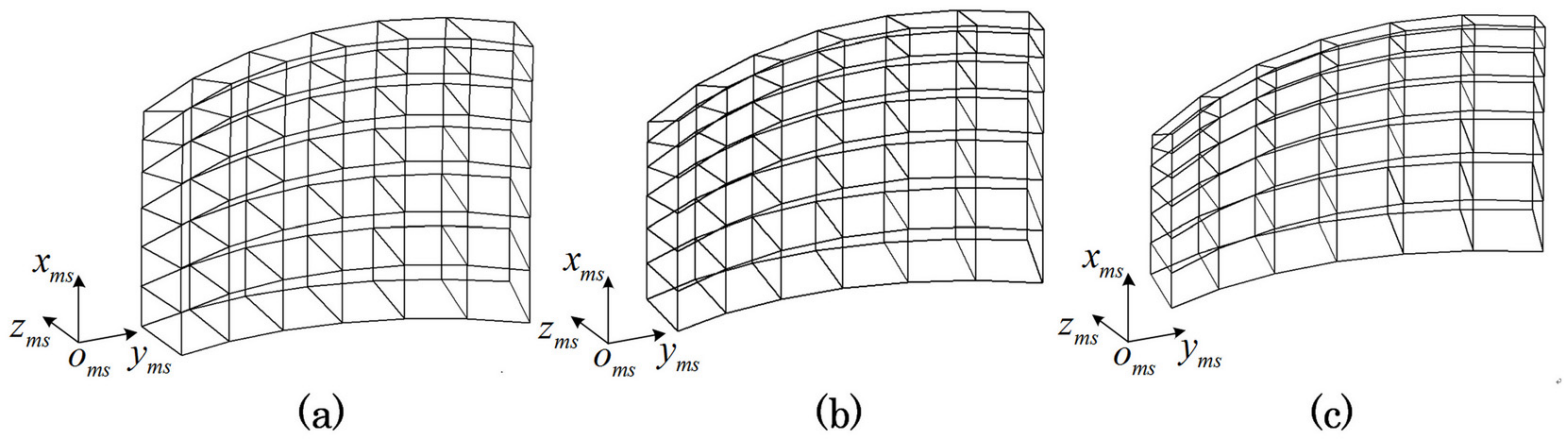
The spatial distribution of feature point. **(a)Roll Angle: 0°~10° (b) Roll Angle: 10°~20° (c) Roll Angle: 20°~30°**. In order to observe conveniently, the simulate curve has been divided into three parts by the roll angle. The other two angle of the curve are yaw angle from 0° to 60° and pitch angle from 0° to 60°. The angle between adjacent points is 10°.

The feature point of different positions filled the moving space, and the spatial distribution of them is simulated. Part of the results are displayed in Fig 6. Each intersection point on the curve corresponds to a feature point. The space surrounded by the curve, which is filled with the feature points, is parameterized by the angle information of feature points.

As the feature points in Fig 6 rotate around the three axes o_mx_-x_ms_, o_mx_-y_ms_, and o_mx_-z_ms_ simultaneously, the trajectories of them beyond description. Fig 6 is different form Fig 4. Scaling down the trajectories in Fig 6, and the scaled trajectories turn into the non-coplanar arcs. The biggest difference between Figs 4 and 6 is that the feature points are used in different ways. Fig 4 focuses on the solid arc that is part of the dotted circle while Fig 6 focuses on the moving space that filled with the feature points. The moving space in Fig 6 is subdivided into small fragments by the curve mesh. The central point of fragment is selected as the control point, and the measuring point is constrained by the control point in the same fragment. Then the implementation of GCPC follows the two steps: the creation of control points and the calibration of measuring point.

### The Creation of Global Control Points

**Fig 7:**
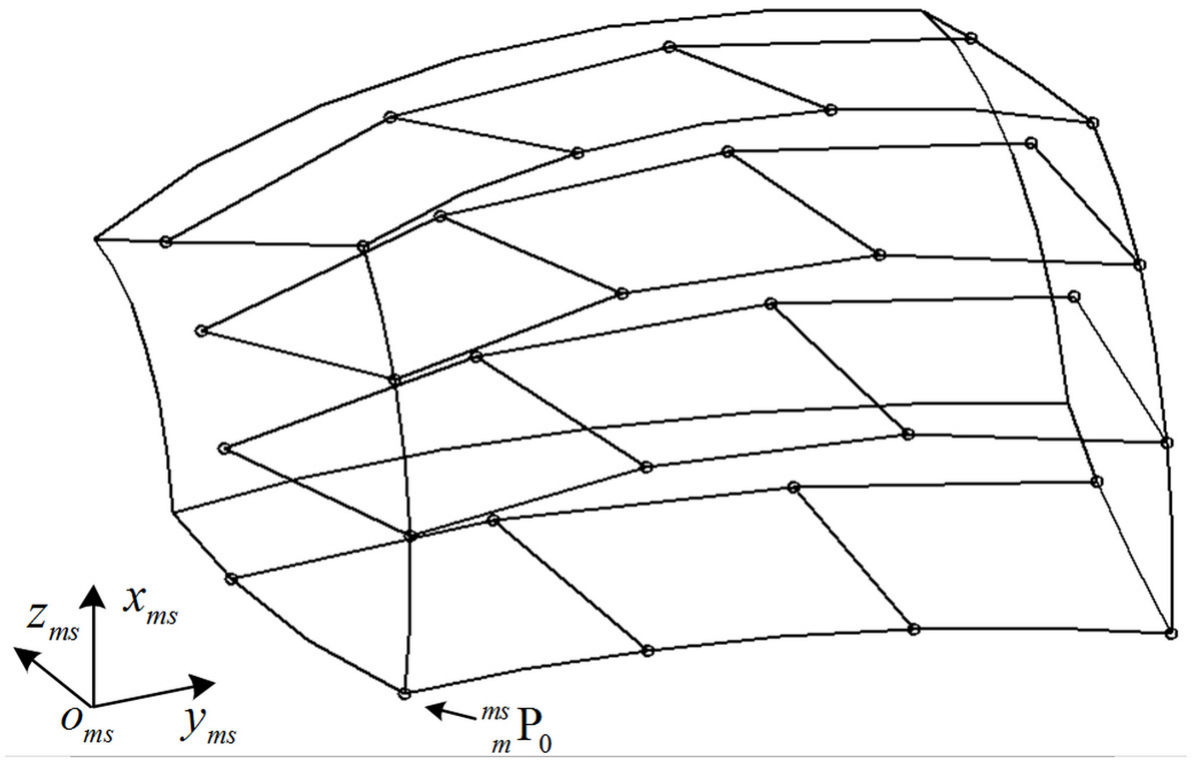
The spatial distribution of initialized control points. The range of curve is the same with that of Fig 6, and it is an eighth of the moving space which is symmetrical around ${}_{m}^{ms}{\text{P}}_{0}$. The initialized control points ‘○’ take control of the surrounding space which is partitioned by adjacent control points.

Given a set of feature point ${}_{m}^{ms}{\text{P}}_{i,j,k}$, (*i*, *j*, *k*) are respectively the number of object’s position in o_mx_-x_ms_, o_mx_-y_ms_, and o_mx_-z_ms_. A sparse point set ${\text{M}}_{I}=\left\{{}_{m}^{ms}{\text{P}}_{i,j,k}\right\}$ is selected as the initialized control points. As the moving space is parameterized by the angle information of feature points, the initialized control points are equally distributed in the angle space. The angle based space is divided as Fig 7.

With the assistance of adjacent points, the points in M_*I*_ divide the moving space into ideal subspaces. The measuring point in the ideal subspace is calibrated by the corresponding control point. But through the analysis of measurement reference, it can be concluded that a system error exists in the O_ms_-X_ms_Y_ms_Z_m_. The axes fitting of o_ms_-x_ms_ and o_ms_-y_ms_ is inaccurate and the three axes are incompletely perpendicular. An angle filter is introduced to eliminate the impact of inaccurate measurement reference. For each point in the moving space corresponding to a pose vector (^*ms*^ *α*, ^*ms*^ *β*, ^*ms*^ *γ)*, the angle between the control point and the measuring point is calculated as:

θti,j,k=arccos(cos(αmsti,j,k)cos(βmsti,j,k)cos(γmsti,j,k)) (10)

where *t* is the number of measuring point. The pose vector (αmsti,j,k,βmsti,j,k,γmsti,j,k) is defined as the following:

Rmsi,j,kt=Rmsi,j,k−1·Rmst (11)

The matrix ^*ms*^ **R**_*i*, *j*, *k*_ is defined as:

{Rmsi,j,k=[h1h2h3]Th1=P2msi,j,kP1msi,j,k→/|P2msi,j,kP1msi,j,k→|h2=P3msi,j,kP0msi,j,k→/|P3msi,j,kP0msi,j,k→|h3=h1×h2 (12)

Rmsi,j,kt is the rotation matrix and turned into Euler angles through Eq (13):

$$R=\left[\begin{array}{ccc} \text{C}\alpha \text{C}\gamma & \text{C}\alpha \text{S}\gamma & -\text{S}\alpha \\ \text{S}\beta \text{S}\alpha \text{C}\gamma -\text{C}\beta \text{S}\gamma & \text{S}\beta \text{S}\alpha \text{S}\gamma +\text{C}\beta \text{C}\gamma & \text{S}\beta \text{C}\alpha \\ \text{C}\beta \text{S}\alpha \text{C}\gamma +\text{S}\beta \text{S}\gamma & \text{C}\beta \text{S}\alpha \text{S}\gamma -\text{S}\beta \text{C}\gamma & \text{C}\beta \text{C}\alpha \end{array}\right]$$ (13)

where C = COS, S = SIN, (*α*, *β*, *γ)* is an abbreviation for (αmsti,j,k,βmsti,j,k,γmsti,j,k).

It is assumed that the total number of M_*I*_ is *M*, and the total number of measuring points is *N*. There are *M* $\theta_{t}^{i,j,k}$ corresponding to the measuring point ${}_{m}^{ms}{\text{P}}_{t}$. Only the point ${}_{m}^{ms}{\text{P}}_{I,J,K}$ which corresponds to the minimum of $\theta_{t}^{i,j,k}$ is selected as the optimized control point. Through this assumption, there are *N* point pairs of optimized control point and measuring point. The frequency of occurrence of the optimized control points is counted, and the cutoff frequency of the angle filter is *N/M*. The candidate control points with lower frequency of occurrence are filtered out. The filtered control point set ${\text{M}}_{II}=\left\{{}_{m}^{ms}{\text{P}}_{i,j,k}\right\}$ is established.

The control space of point in M_*II*_ is extended as the candidate control points are removed. Another subdivision of the control space is employed to improve the calibration capability of point in M_*II*_. The second division is an optimization of the control space by decreasing the angle between two adjacent control points. The subdivided control point set ${\text{M}}_{III}=\left\{{}_{m}^{ms}{\text{P}}_{i,j,k}\right\}$ is formed. The former angle filter can then be reused to the point set M_*III*_, and the final global control point set ${\text{M}}_{IV}=\left\{{}_{m}^{ms}{\text{P}}_{i,j,k}\right\}$ is created.

### The Calibration of Measuring Point

The calibration process is separated into two steps: one is the determination of the pair of control point and measuring point, and the other is the calibration of the pose vector. According to Eq (10), the point pair ${}_{m}^{ms}{\text{P}}_{I,J,K}$ and ${}_{m}^{ms}{\text{P}}_{t}$ is determined by the minimum of $\theta_{t}^{i,j,k}$. Bring the standard pose vector of ${}_{m}^{ms}{\text{P}}_{I,J,K}$ into the following equation:

{αms0t=αms0I,J,K+αmsI,J,Ktβms0t=βms0I,J,K+βmsI,J,Ktγms0t=γms0I,J,K+γmsI,J,Kt (14)

where (αms0t,βms0t,γms0t) is the calibrated pose vector of measuring point ${}_{m}^{ms}{\text{P}}_{t}$.

## The Measurement Procedure

**Fig 8:**
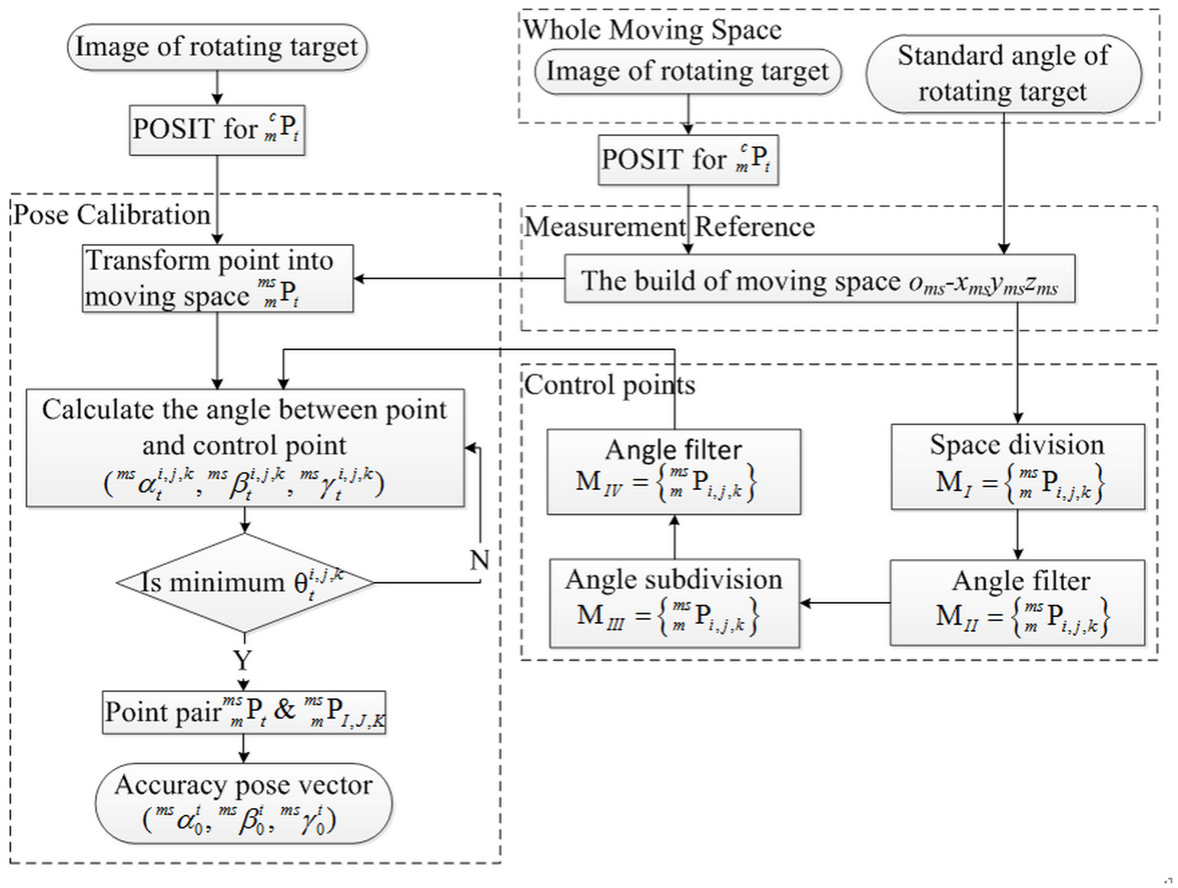
Measurement flowchart.

The measurement procedure of GCPC is shown in Fig 8. GCPC for pose measurement is divided into three steps: the establishment of measurement reference, the creation of global control points, and the calibration of measuring point. The first two steps run only once as the moving space is established.

## Results and Discussion

### Experiment system

For the experiment with real data, an infrared camera is used, and the camera’s field angle is 80°. The internal camera parameters are calibrated [26, 27], and the results are shown in Table 1.

**Table 1:** Internal camera parameters.

**Focal length(mm)**	8.283	**Distortion factor k1**	1.442e-003
**Image center Cx(pixel)**	419.47	**Distortion factor k2**	-4.567e-005
**Image center Cy(pixel)**	246.43	**Distortion factor s1**	1.365e-004
**Nonperpendicularity factor**	1.039	**Distortion factor s2**	-1.902e-004

**Fig 9:**
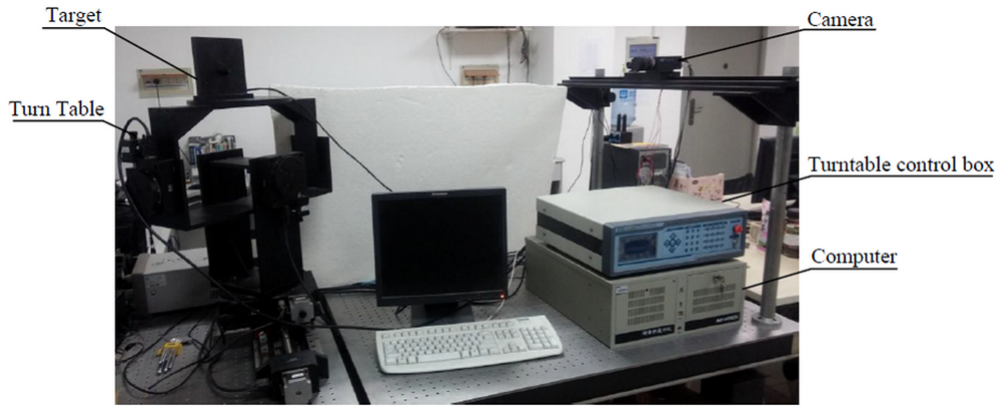
Experiment devices.

The infrared LEDs are selected as positioning feature points, and the relative spatial position of the four feature points is shown in Table 2. Small holes are chosen to be drilled on the support board of target when the target is produced and the LEDs are selected to submerge in the hole. All devices are located on the experiment platform. Fig 9 shows the practical system in laboratory.

**Table 2:** Coordinate of feature points.

Feature point	P_0_	P_1_	P_2_	P_3_
**Coordinate(mm)**	(0.0,60.0,0.0)	(-50.0, -26.603,0.0)	(50, -26.603,0.0)	(0.0,0.0,25.0)

The coordinate is the center of the hole.

### The global control points of GCPC

The range of moving space is -50° to 50° in yaw angle, -50° to 50° in pitch angle, and -30° to 30° in roll angle. The interval angle of sample is 5° at each DOF, and there are 5118 target images within the camera’s field of view.

The images with single DOF rotation can be used to establish the measurement reference. The parameters of measurement reference are shown in Table 3.

**Table 3:** The parameters of measurement reference.

Measurement Reference	Value(mm)
O_ms_-X_ms_	(-0.018,1.000,-0.005)
O_ms_-Y_ms_	(1.000,0.026,-0.014)
O_ms_-Z_ms_	(0.014,0.005,1.000)
O_ms_/mm	(-207.719,-28.601,-889.714)

**Fig 10:**
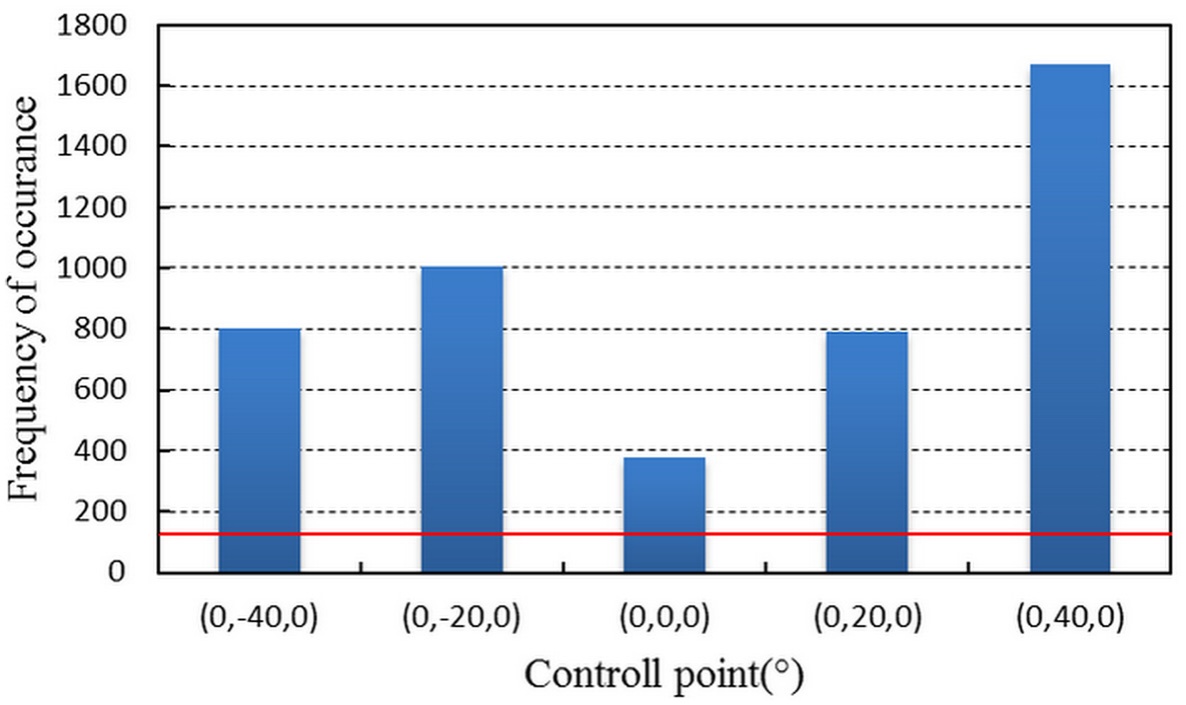
The frequency of occurrence of M_I_. Only the filtered control points are displayed. The red line is the cutoff frequency.

M_I_ = {^*ms*^P_*i*, *j*, *k*_}(*i* = -2,-1,0,1,2, *j* = -2,-1,0,1,2, *k* = -1,0,1) is selected as an initialized control point set. The interval angle is 20° at each DOF. Part of the M_I_ is beyond the camera’s field of view, and the cutoff frequency of M_I_ is 125. Then M_I_ is filtered, and its frequency of occurrence is shown as Fig 10.

**Fig 11:**
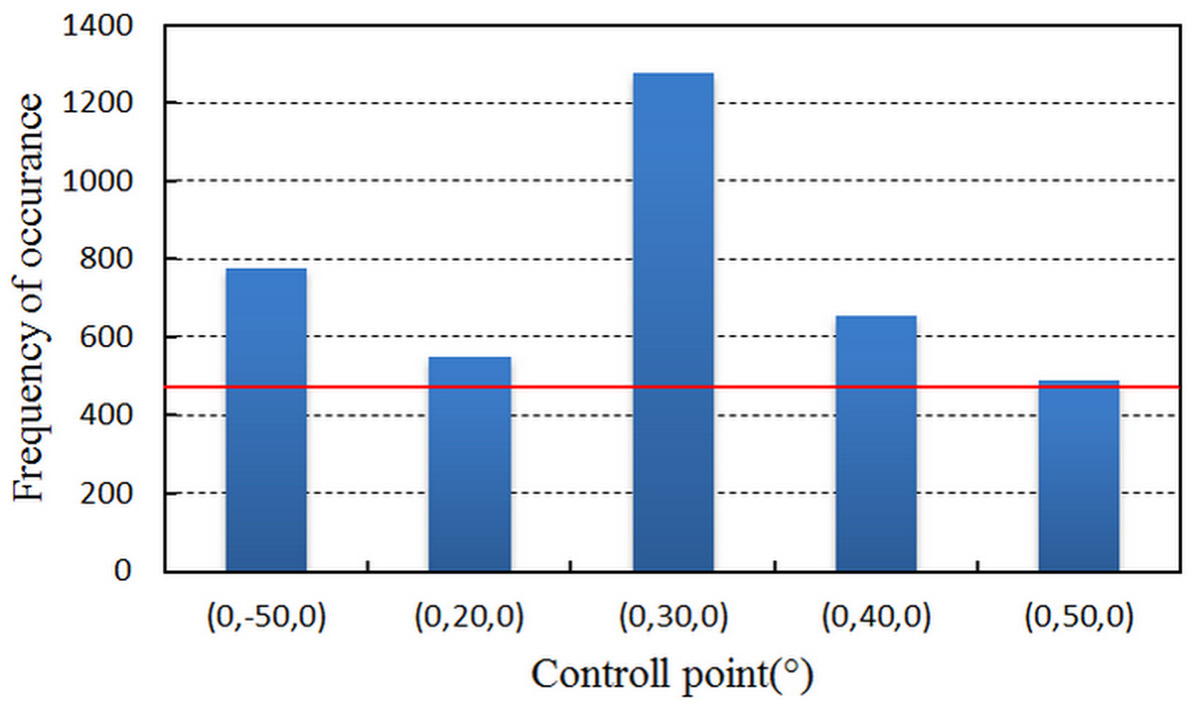
The frequency of occurrence of M_III_. Only the filtered control points are displayed. The red line is the cutoff frequency.

Fig 10 demonstrates that the control points succeed in controlling the space around them, and it is obvious that the uneven distribution is affected by the system error from measurement reference. The control points in Fig 10 constitute control point set M_II_. According to the statistical result, the control space of the points in M_II_ is expanding considerably. Then the points in M_II_ are subdivided by decreasing the interval angle to 10°. The new control points are grouped into control point set M_III_. The cutoff frequency of M_III_ is 466. As the angle filter is performed on M_III_, the statistical results are shown in Fig 11.

From the frequency of occurrence in Fig 11, the final control point set M_IV_ is established. The point set M_IV_ is formed by the points in Table 4.

**Table 4:** Final control point set M_IV_.

Number	Pose Vector(°)	P_0_(mm)	P_1_(mm)	P_2_(mm)	P_3_(mm)
1	(0.0,50.0,0.0)	(-295.064,-30.208,190.964)	(-240.395,-80.301,124.172)	(-238.193,19.679,125.248)	(-275.545,-29.826,128.965)
2	(0.0,40.0,0.0)	(-322.390, -30.019,134.765)	(-257.091,-80.084,78.695)	(-255.045,19.922,79.491)	(-292.506,-29.788,77.046)
3	(0.0,30.0,0.0)	(-341.116,-29.990,82.450)	(-266.845,-80.080,39.182)	(-265.021,19.930,39.816)	(-301.423,-29.853,30.985)
4	(0.0,20.0,0.0)	(-350.089,-30.189,25.842)	(-269.088,-80.194,-3.034)	(-267.478,19.812,-2.048)	(-301.602,-29.93,-17.44)
5	(0.0,-50.0,0.0)	(-152.237,-30.124,-300.785)	(-96.726,-80.113,-234.471)	(-97.415,19.865,-233.586)	(-94.743,-29.775,-270.458)

The pose measurement results which respectively correspond to the four control point sets M_I_, M_II_, M_III_, and M_IV_ are compared in the next section.

### Pose measurement results

**Fig 12:**
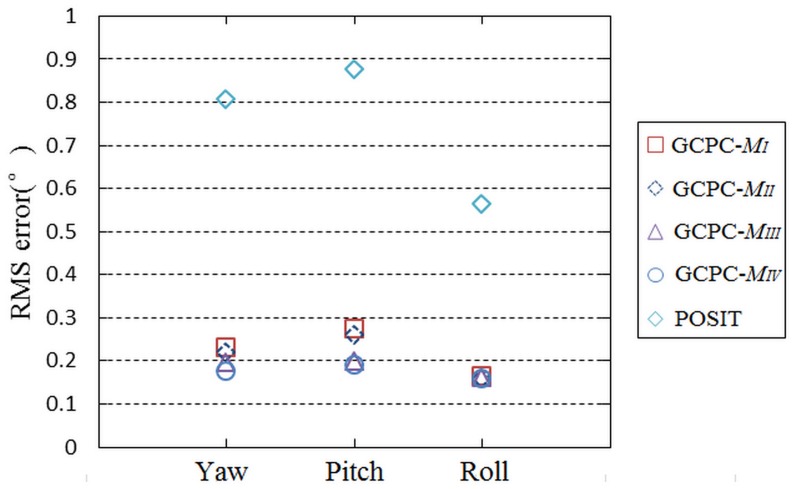
The RMS error of the results. The x axis is used to distinguish the yaw angle, pitch angle, and roll angle.

In order to prove the role of GCPC, the pose measurement of measuring target in the whole moving space is accomplished. The moving space has been established by the O_ms_-X_ms_Y_ms_Z_ms_. The gathered data are transmitted into POSIT and GCPC, and the pose measurement results are analyzed. The control point sets M_I_, M_II_, M_III_, and M_IV_ are respectively adopted by GCPC. The root mean square(RMS) error of GCPC and POSIT are displayed in Fig 12.

By comparing the results of GCPC and those of POSIT, it is obvious that the measurement accuracy of GCPC is higher than that of POSIT in the whole moving space. The comparisons of the four control point sets demonstrate that the creation of global control points is effective.

**Fig 13:**
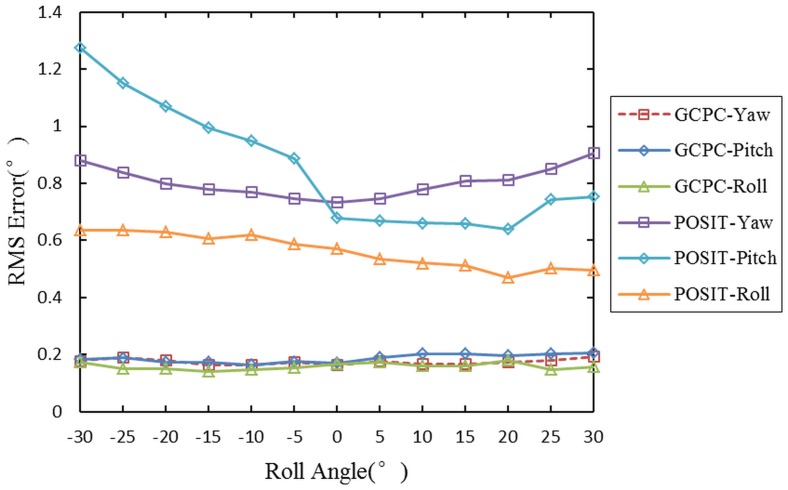
The RMS error of the surfaces of angle. The x axis represents the third angle.

In order to test the error distribution of GCPC, the measuring points are classified into the surface of angle determined by three angles. The range of the first two angles are respectively -50° to 50° in yaw angle, -50° to 50° in pitch angle. The third angle changes from -30° to 30°. The RMS error of the surfaces of angle are shown in Fig 13.

The RMS error of GCPC is far less than that of POSIT. The former is stable and reduced to 0.2° while the latter fluctuates along the roll angle and reaches 1.2°. The steep trend of POSIT demonstrates that the measurement error mentioned earlier exits in the pose measurement process, and the gentle trend of GCPC proves that the measurement error is calibrated successfully in the whole moving space.

**Fig 14:**
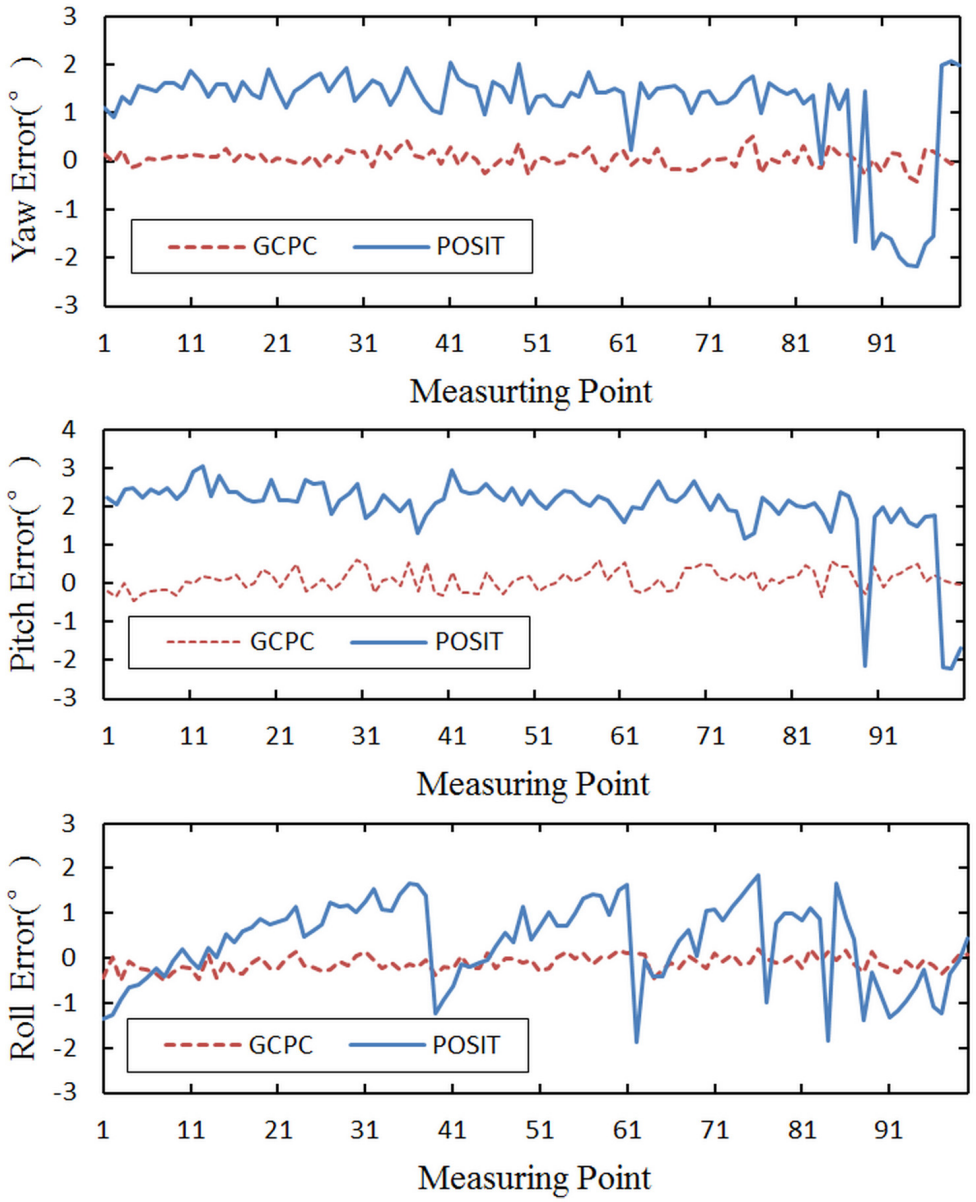
The accuracy comparison between GCPC and POSIT.

The above data analysis is based on the RMS error, and 100 measuring points with the maximal errors are selected. The optimization of GCPC to the measuring points is shown in Fig 14.

Through analysis of Fig 14, it is evident that the measurement error is reduced by GCPC. The control point error which is the primary source of the measurement error is eliminated successfully, and the error curve which fluctuates around zero is caused by the control space error.

## Conclusions

In this paper, GCPC is developed to optimize the pose measurement error. The control point error is redefined to be the primary source of measurement error, and calibrated by the corresponding global control point. The control space error has less impact on the pose measurement, and minimized by the subdivision of control space. Both of the creation of global control points and the calibration of pose measurement have been confirmed by experiment. The experiment results show that the pose measurement process is calibrated by the global control points successfully. To sum up, GCPC improves the accuracy of pose measurement.

## Supporting Information

S1 Dataset Camera captured dataset. This excel contains the capture data used as the basis for the pose measurement solution described in the manuscript. The data is given by means of image coordinate.
(XLSX)
